# Modification of Histone Acetylation Facilitates Hepatic Differentiation of Human Bone Marrow Mesenchymal Stem Cells

**DOI:** 10.1371/journal.pone.0063405

**Published:** 2013-05-02

**Authors:** Xuejun Dong, Ruolang Pan, Hui Zhang, Chao Yang, Jianzhong Shao, Lixin Xiang

**Affiliations:** 1 Shaoxing People's Hospital, Shaoxing Hospital of Zhejiang University, Shaoxing, Zhejiang Province, P. R. China; 2 College of Life Sciences, Zhejiang University, Hangzhou, P. R. China; 3 Key Laboratory for Cell and Gene Engineering of Zhejiang Province, Hangzhou, P. R. China; 4 School of Laboratory Medicine and Life Science, Wenzhou Medical College, Wenzhou, Zhejiang Province, P. R. China; Peking University Health Science Center, China

## Abstract

The multi-potentiality of mesenchymal stem cells makes them excellent options for future tissue engineering and clinical therapy, including liver injury. In this study, we investigated the effects of valproic acid (VPA), a direct inhibitor of histone deacetylase (HDAC), on the hepatic differentiation of human bone marrow mesenchymal stem cells (BMMSCs). The cells were found to differentiate into a more homogeneous hepatocyte-like population when pretreated with 5 mM VPA for 72 h. The expression of liver-specific markers was significantly upregulated in the VPA-treated group at the mRNA and protein levels. VPA treatment also significantly enhanced the hepatic functions of the differentiated cells, including glycogen storage, cytochrome P450 activity, AFP and ALB synthesis, and urea production. Further analysis showed that treatment with 5 mM of VPA for 72 h greatly improved the histones H3 and H4 acetylation. These results demonstrated that VPA could considerably improve the hepatic differentiation of human BMMSCs, probably because the chromatin-acetylated state changes upon VPA treatment through its HDAC inhibitory effect. Thus, this study provides a direct research model for producing human hepatocytes for clinical purposes.

## Introduction

Severe acute liver failure carries high mortality rate ranging from 60% to 90%. Orthotopic liver transplantation, its only therapeutic option, is limited by the shortage of suitable donors. Therefore, hepatocyte transplantation has emerged as a promising alternative treatment. However, the application of this procedure has been hampered by the difficulty of obtaining freshly isolated hepatocytes [1]. Accordingly, the effective induction of functional hepatocytes from autologous non-hepatic sources would be a significant improvement.

Bone marrow mesenchymal stem cells (BMMSCs), which are readily acquired, could capably differentiate into the mesoderm cell lineages, such as the osteoblasts, chondrocytes, and adipocytes [2]–[4]. Recently, numbers of studies also demonstrated that BMMSCs had plasticity for multiple cell lineages, such as neurons, epidermal-like cells, and hepatocytes, both *in vivo* and *in vitro* using animal models [5]–[7]. The multi-potentiality of BMMSCs, together with their autologous nature, relatively lesser ethical concerns, and lower incidence rates of rejection, makes these cells an excellent option for future tissue engineering and clinical therapy [8]–[12]. The development of effective protocols for the hepatic differentiation from BMMSCs will be beneficial not only in obtaining a better understanding of hepatogenesis but also for further clinical purposes. Several hepatic differentiation systems of BMMSCs from different origins have been established in the past decade [3], [6], [7], [13]. Nevertheless, a more rapid and effective method for hepatic specification is still required before BMMSCs become the therapeutic choice for liver failures.

HDAC inhibitors, such as VPA, sodium butyrate (NaB), and trichostatin A (TSA), exhibit profound therapeutic effects in preclinical tumor tests with their ability to regulate the proliferative and apoptotic specific genes expressions [14]–[17]. In a previous study, we found that exposure of mouse BMMSCs to VPA considerably improved the BMMSCs' hepatic differentiation and promoted their adaptation to the injured liver [18]. This finding suggested that VPA has potential applications in human translational medicine. To provide direct evidence, we purposefully investigated the role of VPA in hepatic differentiation of human BMMSCs. The results clearly demonstrate the profound effect of VPA on the hepatic differentiation of human BMMSCs. Thus, this study aims to provide a direct research model for the production of human hepatocytes for clinical purposes.

## Materials and Methods

### Isolation and Culture of Human BMMSCs

Human BMMSCs from the bone marrow of healthy adult volunteers were isolated according to a previously reported [7] method with some minor modifications. Written informed consent was obtained from each participant, and the study was approved by the ethics committees of Shaoxing People's Hospital and Shaoxing Hospital of Zhejiang University. Bone marrow mononuclear cells were isolated via the Ficoll – Hypaque (density, *D* = 1.077±0.002 g/mL; Sigma, St. Louis, MO, USA) density gradient centrifugation (2200 rpm/min, 20 min at 4°C). The isolated cells were cultured in Iscove's modified Dulbecco's medium (IMDM; Sigma), supplemented with 10% fetal bovine serum (Hyclone, Rockville, MD, USA), 100 U/mL of penicillin (Gibco BRL, Rockville, MD, USA), and 100 mg/mL of streptomycin (Gibco BRL) at 37°C in 5% CO_2_. After three days, the non-adherent cells and debris were removed, whereas the adherent cells were further cultured. Cells were digested using 0.25% trypsin-EDTA (Sigma) after being grown into 70% to 90% confluence and seeded into glass flasks at a cell density of 4×10^4^ per cm^2^ as the first passage. The cells at third passage were used for functional characterization and hepatic differentiation studies, and the cultures were maintained by changing the medium every three days.

### Characterization of Human BMMSCs

Cells were characterized by their mesenchymal lineage differentiation potentials as previously described [18] with some modifications: To induce osteogenic differentiation, cells were cultured in Iscove's modified Dulbecco's medium (IMDM) supplemented with 10 mM β-glycerophosphate, 50 mg/mL ascorbic acid and 10^−7^ M dexamethasone (Dex). After three weeks, differentiated cells were examined by alkaline phosphatase (ALP), Alizarin red and Von Kossa staining. Chondrogenic differentiation was achieved using a chondrocyte-inductive medium (IMDM supplemented with 10 ng/ml transforming growth factor beta and 50 µg/mL vitamin C). After three weeks of culture, the differentiated cells were stained with alcian blue, and observed via phase-contrast light microscopy. Adipogenic differentiation was induced using an adipocyte-inductive medium (IMDM supplemented with 50 µg/mL insulin and 10^−7^ M Dex). One week after induction, the intracellular fat droplets were detected via phase-contrast light microscopy and chemical staining using Oil Red O.

### Induction of Hepatic Differentiation

**Figure 1:**
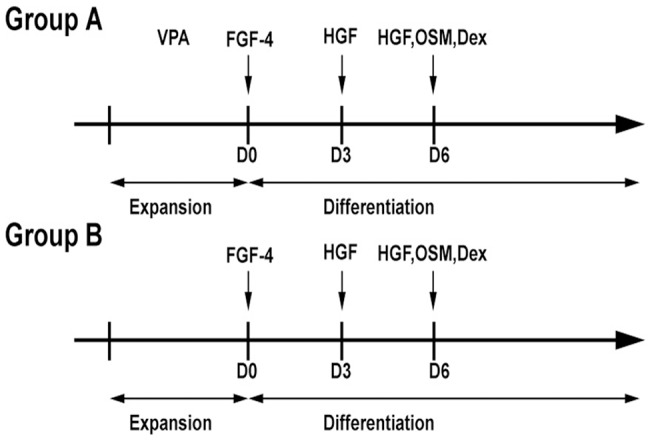
Schematic presentation of the differentiation protocol.

The human BMMSCs at third passage were divided into two groups (Fig. 1). Briefly, in Group A, the cells were pre-treated with 5 mM VPA for 72 h and then induced using cytokines (20 ng/mL FGF-4, 30 ng/mL HGF, 20 ng/mL OSM, 1× ITS, and 40 μg/mL Dex; R&D Systems, Abingdon, U.K.) at specified time points, in a manner that closely resembles the secretion pattern during liver ontogeny. In Group B, the cells were treated only with cytokines to serve as the control group. Hepatic differentiation was characterized based on cell morphology, as observed under a Zeiss phase contrast microscope (Axiovert 40 CFL, Carl Zeiss Inc., Jena, Germany), the expression levels of hepatic genes and proteins, and a number of functional observations.

### Hepatic Gene Expression Analysis

The expression analysis of several hepatic genes, AFP, HNF3β, and ALB, was performed to determine the extent of hepatic differentiation after VPA pre-treatment for 7 and 21 days. Total RNA was isolated from the differentiated cell, and the complimentary DNA was synthesized via reverse transcription. The PCR primers and conditions are shown in Table 1. The number of PCR cycles for detecting each gene expression was determined to semi-quantify the amount of mRNA within a linear range. The PCR products were separated through 1.2% agarose gel electrophoresis and visualized under UV illumination after being stained with ethidium bromide. The gel images were captured and analyzed by the Kodak Gel Logic 200 Imaging System (Kodak Molecular Imaging Systems, USA). The expression levels of the genes were quantified by real-time PCR (RT-PCR) using Mastercycler ep realplex (Eppendorf, Hamburg, Germany) and Real-Time Detection System software. The mRNA expression was normalized to endogenous β-actin mRNA.

**Table 1:** Primers and Annealing Temperatures Used for RT-PCR.

Gene	Primers(5′–3′)	Product size	Annealing
β-actin-F	ACACCTTCTACAATGAGCTG	800 bp	58°C
β-actin-R	CTGCTTGCTGATCCACATCT		
AFP-F	TGCAGCCAAAGTGAAGAGGGAAGA	217 bp	68°C
AFP-R	CATAGCGAGCAGCCCAAAGAAGAA		
HNF3β-F	CGTTCCGTCCCAAACAGAG	410 bp	58°C
HNF3β-R	TAAAGCACGCAGAAACCATA		
ALB-F	TGCTTGAATGTGCTGATGACAGGG	162 bp	58°C
ALB-R	AAGGCAAGTCAGCAGGCATCTCATC		

### Immunofluorescence Staining

The differentiated cells were fixed in 4% paraformaldehyde, permeabilized with cold methanol, and incubated with the primary antibodies, rat anti-HNF3β and goat anti-ALB (Biodesign, Saco, Maine, USA). Then, the secondary antibodies, namely, fluorescein isothiocyanate (FITC)-conjugated rabbit anti-goat IgG and tetramethylrhodamine isothiocyanate (TRITC)-conjugated goat anti-rat IgG (Santa Cruz Biotechnology, Santa Cruz, CA, USA), were used according to the manufacturer's instructions. After being further washed or counterstained with 4′,6-diamidino-2-phenylindole (DAPI), the samples were photomicrographed under a confocal laser-scanning microscope (LSM 510; Carl Zeiss Inc., Jena, Germany).

### Periodic acid – Schiff (PAS) Staining for Glycogen

The differentiated cells were fixed in 95% alcohol for 10 min. The samples were then oxidized in 1% periodic acid for 5 min and rinsed three times in deionized water (dH_2_O) before being treated with Schiff's reagent for 15 min and rinsed in dH_2_O for another 5 min. Finally, the preparations were assessed under a light microscope.

### Detection of Cytochrome P450 Activity

The activity of cytochrome P450 (CYP), specifically CYP2B, in the differentiated cells was determined as previously described. Briefly, phenobarbital (PB; Wako Pure Chemical) was used as a CYP inducer. To enhance CYP2B expression, the cells were incubated in a basal medium containing 2 mM of PB, which was renewed every day for three consecutive days. The cells were washed twice with phosphate-buffered saline (PBS) and then treated with 5 mM of 7-ethoxyresorufin (diluted in Medium A) as its substrate for 3 h at 37°C. The 7-Ethoxyresorufin-O-deethylase (EROD) activity catalyzed by CYP2B was detected using a fluorescence detector (excitation at 355 nm; emission at 581 nm).

### Detection of AFP and ALB Synthesis

The amounts of AFP and ALB in the differentiated cells after 0, 7, 14, and 21 days were measured via enzyme-linked immunosorbent assay (ELISA) [19]. Briefly, 96-well plates were coated with cell extracts and incubated overnight at 4°C. The plates were washed thrice with PBS containing 0.05% Tween-20 and blocked with 3% BSA for 1 h at 37°C. Then, the anti-ALB or anti-AFP antibodies (Biodesign, Saco, Maine, USA) were added to the respective wells, and the plates were incubated for 1 h at 37°C. The plates were washed, and goat anti-rabbit IgG (Santa Cruz Biotechnology, Santa Cruz, CA, USA) were added. The plates were then incubated for another hour at 37°C and washed again before the peroxidase-conjugated streptavidin rabbit IgG was added. Finally, the color was developed by adding the 3,3′,5,5′-tetramethylbenzidine (TMB) substrate solution. The plates were analyzed on an ELISA reader at 450 nm.

### Evaluation of Urea Production

The urea concentrations in the cell cultures were determined by a colorimetric assay (640-1; Sigma-Aldrich) following the manufacturer's instructions and analyzed using BioPhotometer 6131 (Eppendorf, Hamburg, Germany). After being washed extensively with PBS, the cells were incubated in 2 mL of basal medium containing 5 mM NH_4_Cl for 24 h at 37°C. The urea concentrations in the supernatant were then measured after incubation.

### Histone Acetylation Analysis

The level of histone acetylation in VPA-treated cells was analyzed via immunofluorescence staining and Western blot. Briefly, the cells were exposed to 5 mM of VPA for 72 h. Immunofluorescence staining was performed using the anti-acetylated histone H3 (mouse monoclonal to acetyl K9 in histone H3; Abcam, UK) and anti-acetylated histone H4 (rabbit monoclonal to acetyl K8 in histone H4; Abcam, UK) antibodies, as described in the abovementioned method. For the Western blot analysis, the protein extracts from both VPA-treated and untreated cells were electrophoresed and subsequently blotted. Blots were incubated with antibodies for the acetylated histones H3 and H4 and then with secondary horseradish peroxidise-conjugated antibody before they were visualized using the enhanced chemiluminescence.

### Statistical Analysis

Statistical analysis was performed using SPSS version 16.0, and the data were expressed as mean ± standard deviation. The differences between the values were determined using the independent sample t-test. A value of P<0.05 was considered to be significant, whereas P<0.01 was highly significant.

## Results

### Characterization of culture-expanded human BMMSCs

**Figure 2:**
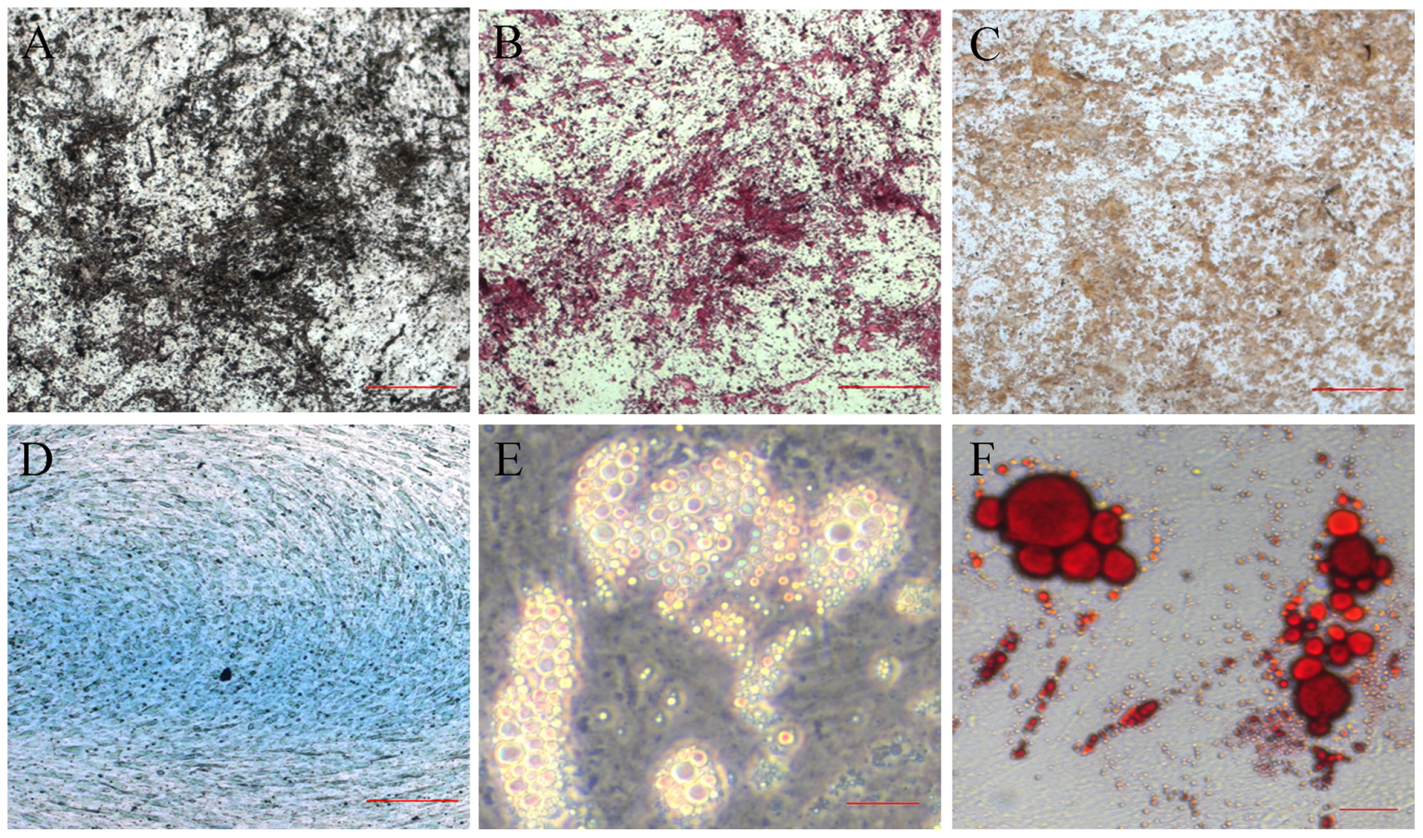
Characterization of human BMMSCs. (A–C) Cells cultured in osteoblast-inductive medium for three weeks, visualized with ALP (A), alizarin red (B) and von Kossa (C) staining to show human BMMSCs that differentiated into osteogenic cells. Scale bar, 100 μm; (D) Chondrogenic differentiation in chondrocyte-inductive medium after three weeks, with the differentiated cells examined via alcian blue Staining. Scale bar, 50 μm; (E, F) Adipogenic differentiation of human BMMSCs induced by adipocyte-inductive medium. One week after induction, intracellular fat droplets were detected via phase-contrast light microscopy (E) and Oil Red O staining (F). Scale bar, 10 μm.

Using Ficoll – Hypaque density gradient centrifugation, mononuclear cells were isolated from the bone marrow of healthy adult volunteers. After three days, adherent cells with fibroblast shapes could be observed. To exclude the possible pollution of hematopoietic cells, the fibroblastic cells were selected according to their plastic adherence and continuous passage for at least three times before further use. The differentiation potentials of these cells were functionally identified to examine whether they have the characteristics of the BMMSCs. Alkaline phosphatase, alizarin red S, and von Kossa staining showed that the cells had the potential to differentiate into osteogenic cells (Fig. 2A–C). Alcian blue staining indicated that the cells could also differentiate into chondrocytes (Fig. 2D). When differentiated on adipogenic media, cells with lipid-producing vacuoles could be detected (Fig. 2E–F). The above results showed a cell population that is devoid of hematopoietic cells and macrophages but functionally and phenotypically equivalent to genuine BMMSCs.

### Hepatic morphological observation

**Figure 3:**
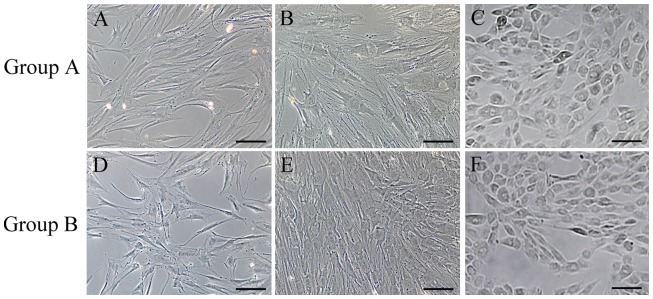
Light microscopy of the hepatic differentiation of human BMMSCs. (A and D) The morphological changes of differentiated cells in Groups A (VPA+) and B (VPA−) were observed under a Zeiss phase contrast microscope at days 3; (B and E) Morphological changes of differentiated cells at days 14; (C and F) Morphological changes of differentiated cells at days 21. Scale bar, 20 μm.

To evaluate whether VPA treatment could improve the hepatic differentiation of human BMMSCs, we compared the morphological changes of treated and untreated cells during hepatic differentiation at designated time points (Fig. 3). The results showed that hepatocyte-like epithelioid cells appeared at day 14 in the VPA-treated group, although most of the cells were still surrounded by spindle-shaped cells (Fig. 3B). In contrast, only a few hepatocyte-like epithelioid cells could be observed in the untreated cells at day 14, and most cells still displayed a fibroblastic profile (Fig. 3E). This result indicates that VPA-treated cells own an earlier switch of hepatic differentiation compared to the control. After 21 days, a more homogeneous hepatocyte-like population was developed in the VPA-treated group. The number of cells exhibiting a polygonal shape with a clear round nucleus in this group (Fig. 3C) was obviously higher than that in the control group (Fig. 3F). These observations indicate that VPA treatment can significantly improve the hepatic differentiation of human BMMSCs.

### Hepatic gene expression analysis

**Figure 4:**
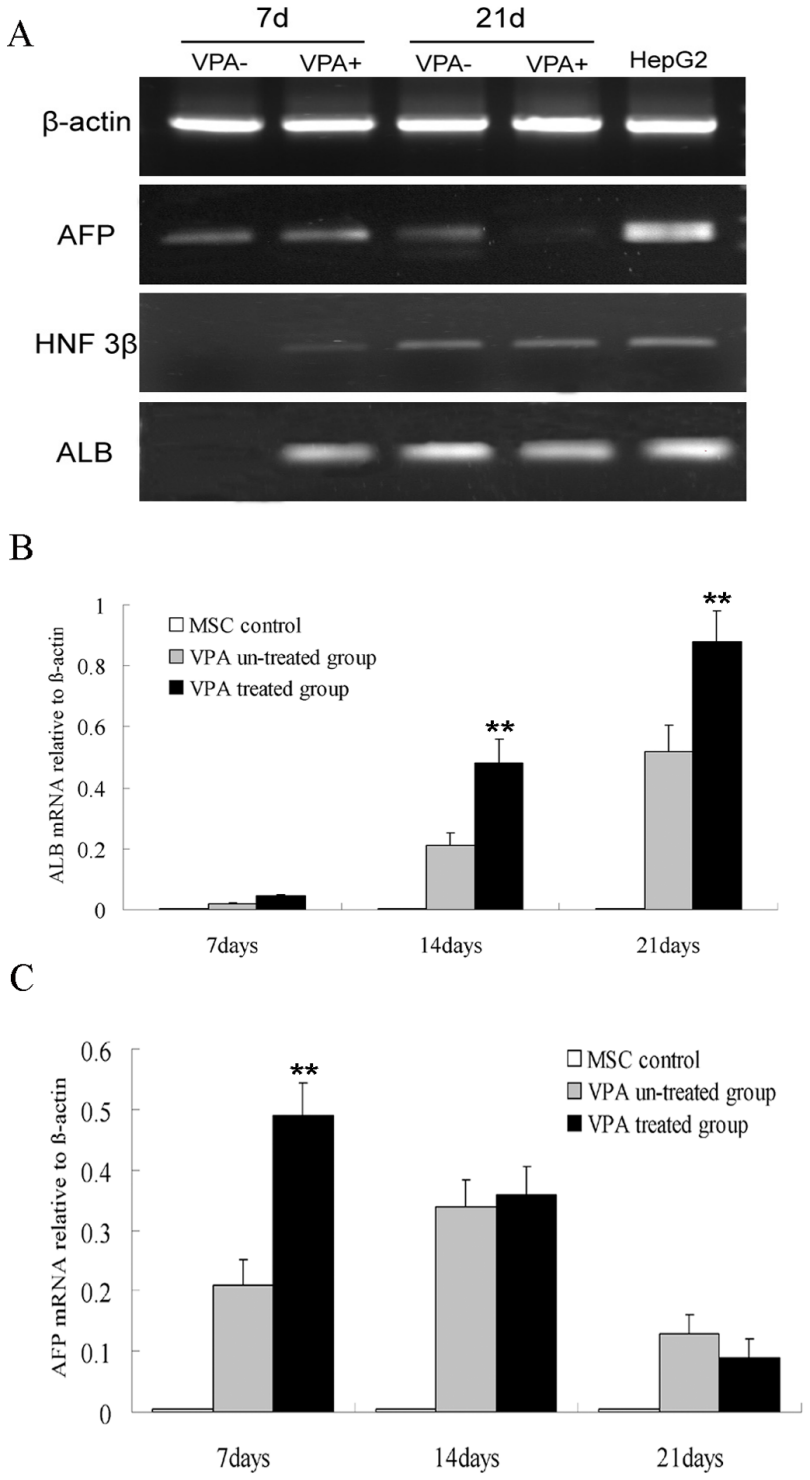
Hepatic gene expressions of differentiated cells at the mRNA level. (A) PCR results showing the time-dependent expression of AFP, HNF3β, and ALB in both groups during hepatic differentiation; (B) Expression level of ALB as confirmed by RT-PCR (**P<0.01 versus VPA untreated group); (C) Expression level of AFP detected by RT-PCR (**P<0.01 versus VPA untreated group).

**Figure 5:**
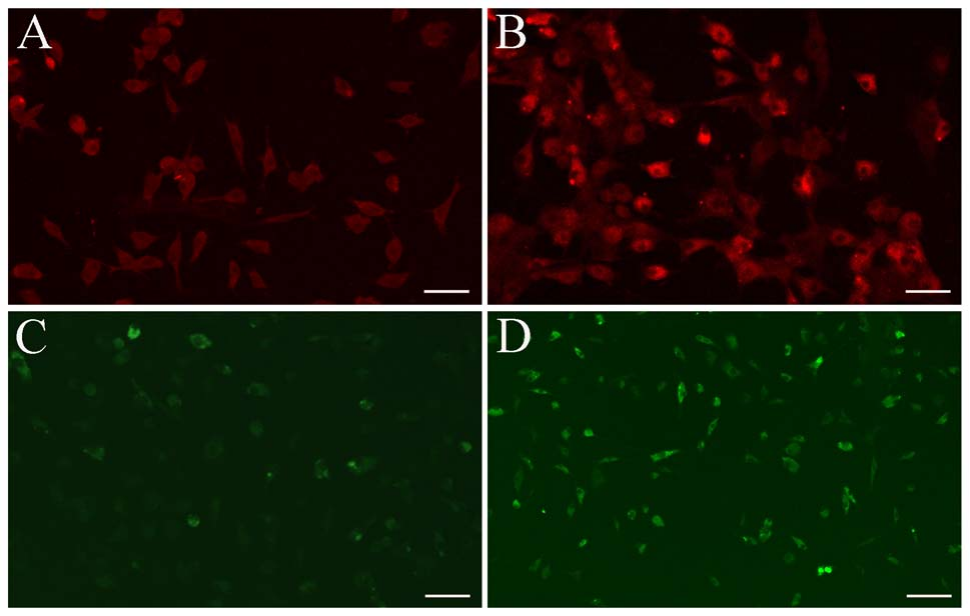
Immunofluorescence staining of hepatocyte-specific markers in the differentiated cells. (A, B) Differentiated cells were stained with a specific antibody for HNF3β in untreated (A) and VPA-treated groups (B); (C, D) Immunofluorescence of ALB showed similar results in the control (C) and VPA-treated cells (D). Scale bar, 20 μm.

The expressions of liver-specific markers, AFP, HNF3β, and ALB, were analyzed at both the mRNA and protein levels during hepatic differentiation. PCR analysis demonstrated that the expressions of AFP, HNF3β, and ALB increased with time in both the VPA-treated and untreated groups during cell differentiation with different levels (Fig. 4A). During the early stages (within seven days) of differentiation, much higher transcript levels of AFP and HNF3β (two early hepatic differentiation markers) could be detected in the VPA-treated groups. This result indicates that hepatic differentiation in the VPA-treated group occurred earlier than in control group. The difference on mRNA expression levels of AFP between the two groups was further confirmed by RT-PCR analysis. Moreover, RT-PCR analysis showed that mRNA expression levels of ALB (the mature hepatic differentiation marker) was significantly (P<0.01) up-regulated in the VPA-treated group from day 14 to the later stages of differentiation (day 21) (Fig. 4B and 4C). Immunofluorescence staining also showed that the expression levels of HNF3β and ALB proteins in the VPA-treated cells were higher than those in the control cells (Fig. 5), which is consistent with the results obtained at the mRNA levels.

### Functional evaluation of the differentiated cells

**Figure 6:**
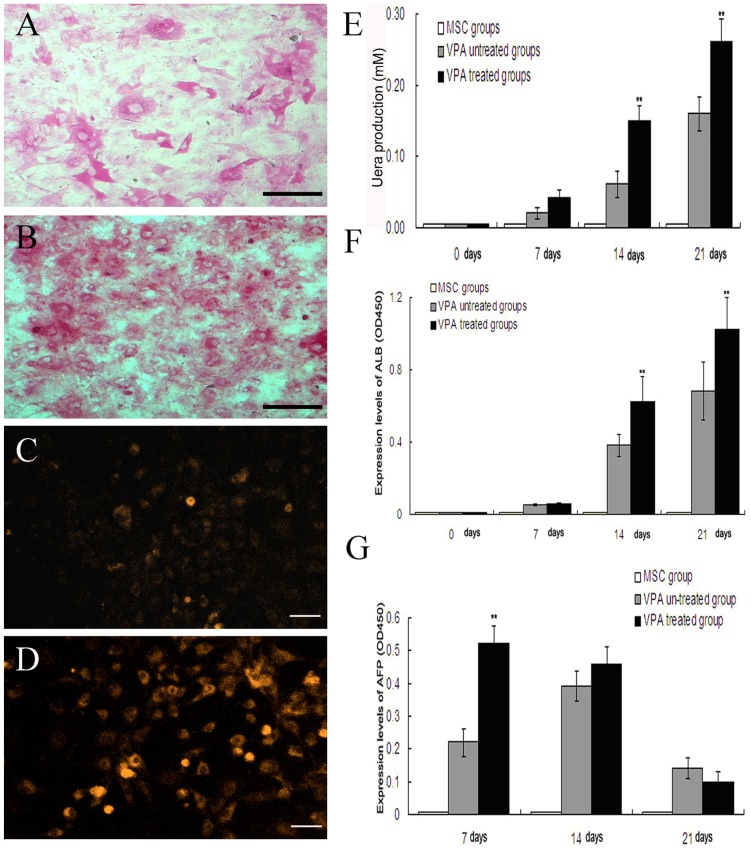
Functional evaluation of differentiated cells. (A, B) PAS staining of untreated (A) and VPA-treated (B) cells; (C, D) Expression of functionally active *CYP2B* in untreated (C) and VPA-treated (D) cells; (E-G) Urea production, *ALB* and *AFP* syntheses at various time points during differentiation (**P<0.01 versus VPA untreated group). Scale bar, 50 μm.

The hepatic functional characterization of the differentiated cells, including glycogen storage, cytochrome P450 activity, AFP and ALB synthesis, and urea production, were further examined. The results clearly showed that the VPA-treated cells were more sensitive to PAS staining and more abundantly expressed the functionally active CYP2B compared with the untreated cells (Figs. 6A–D). Furthermore, the urea production, and AFP and ALB synthesis abilities were determined at various time points during hepatic differentiation. The results demonstrate that urea production in both groups could be detected at day 7, increasing over time. However, at days 14 and 21, urea production in the VPA-treated cells was significantly up-regulated (P<0.01) compared with that in the control group (Fig. 6E). Similarly, the synthesis of ALB could be detected in both groups at day 14 and was then up-regulated by day 21. However, the synthesis of ALB was more pronounced in the VPA-treated group than in the control group at these two time points (Fig. 6F). In contrast, AFP synthesis started at the early stage of differentiation and reached its peak at day 7 with a significant (P<0.01) up-regulation in the VPA-treated groups. Then, it decreased in the following days. This result suggests that VPA might significantly facilitate the early hepatic differentiation of human BMMSCs by up-regulating the early hepatic differentiation genes, including AFP, which in turn, accelerates the maturation of hepatic cells (Fig. 6G). All these functional improvements after VPA pre-treatment indicate that this chemical could dramatically promote the hepatic differentiation of human BMMSCs.

### Histone acetylation analysis

**Figure 7:**
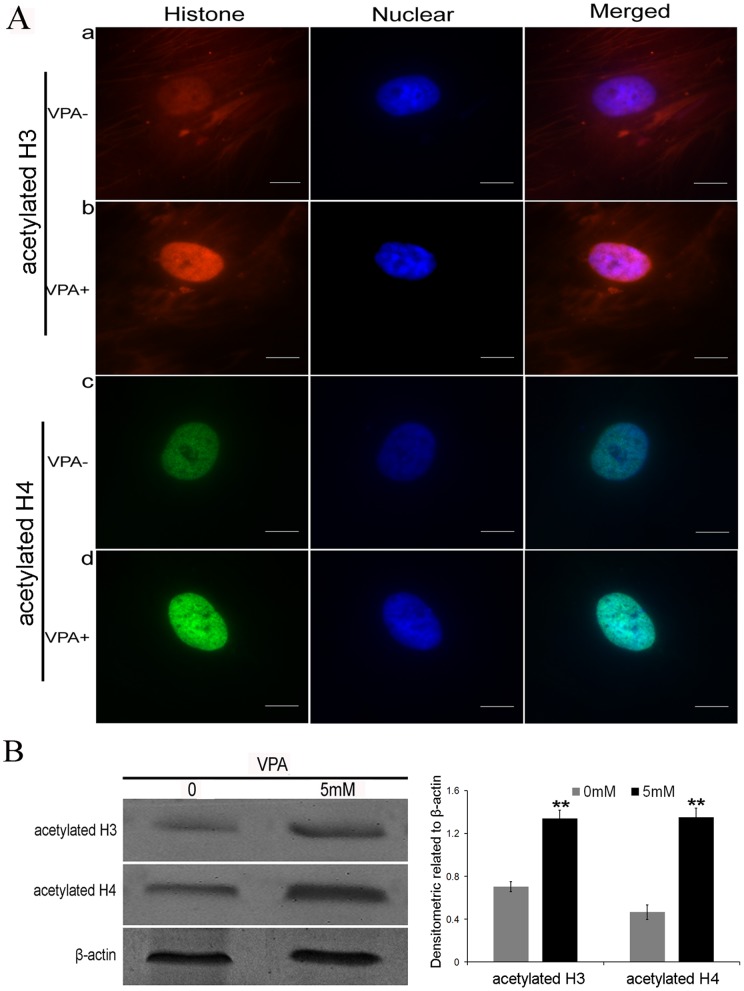
Histone acetylation by immunofluorescence staining and Western blot analysis. (A) Immunofluorescence of acetylated histones H3 and H4 in cell nuclei exposed to 5 mM of VPA for 72 h with counterstaining by DAPI; (B) Western blot analysis showing the significantly up-regulated levels of histone H3 and H4 acetylation after VPA treatment. Densitometric quantifications related to β-actin were analyzed (**P<0.01 versus 0 mM VPA group, n = 5). Scale bar, 20 μm.

To check whether the VPA-mediated hepatic differentiation was accompanied by the alteration of acetylated histones, we evaluated the levels of histone acetylation using immunofluorescence staining and Western blot analysis. Immunofluorescence staining showed a definite increase in the amount of the acetylated histones H3 and H4 in the nuclei of cells exposed to 5 mM of VPA for 72 h (Fig. 7A). Western blot analysis confirmed the observation that the acetylation levels of both histones H3 and H4 were greatly improved after pre-treatment with VPA (Fig. 7B). Therefore, these results suggest that the chromatin acetylated state was changed by VPA through the inhibition of HDAC, which may have global and dominant effects on hepatic differentiation.

## Discussion

The current effective treatments for severe liver injuries are limited; thus, alternative therapeutic approaches are necessary. Multi-potential mesenchymal stem cells, which have fewer ethical concerns and less risk of rejection, are an excellent choice for future stem cell-based liver disease treatment. In recent years, the ability of these stem cells to differentiate into functional hepatocyte-like cells has been demonstrated using different strategies both *in vitro* and *in vivo* [7], [20], [21]. Nevertheless, more efficient strategies for producing enough functional hepatocytes from stem cells for clinical purposes still need to be defined. Most of the recent studies to address this issue were performed on animal models, such as mice, whereas investigations designed to directly use human cells were relatively limited. Thus, more attention should be given to translational studies from the model animals to the application of these technologies on humans. Herein, we showed an improved method for the hepatic differentiation of human BMMSCs by VPA pre-treatment using a well-established system of induced hepatic differentiation. Our results show that a more homogeneous hepatic cell population with stronger functionality could be obtained from VPA-treated cells, indicating significantly increased hepatic differentiation in the present strategy.

Recently, much attention has been given to epigenetic modifications in stem cell differentiation, particularly HDAC-regulated histone acetylation. For example, HDAC inhibitors have been used for neuronal differentiation, cardiomyocyte differentiation from embryonic stem cells, and osteogenic differentiation from adult stem cells [15], [22]–[24]. Our previous study demonstrated that VPA could efficiently stimulate the hepatic differentiation of mouse BMSSCs [18]. However, the effects of the HDAC inhibitor on the hepatic differentiation of human cells had not been well defined. Thus, we addressed this problem in the present study and demonstrated that VPA could also significantly improve the hepatic differentiation of human BMSSCs. Upon further examination, VPA was shown to increase the amount of acetylated histones H3 and H4 in human BMMSCs. The results revealed that the HDAC inhibitor could induce the alteration of global histone acetylation levels, which may be involved in the VPA-induced improvement of the hepatic differentiation of human BMMSCs.

In conclusion, a more efficient system for inducing the hepatic differentiation of human BMMSCs by VPA has been described in this study. The effects of VPA can probably be attributed to the modification of the acetylated state of the genes involved in the hepatic lineage progression. By using human BMMSCs, we established a more direct approach for the application of basic experimentation into clinical practice. Thus, this model could be used not only in further induction of functional hepatocytes for cell-based therapies using BMMSCs but also in the investigation of epigenetic modifications during the generation and development of the human liver.
